# Anatomical Study on Meckel Cave with Endoscopic Endonasal, Endo-Maxillary Sinus, and Endo-Pterygoid Process Approaches

**DOI:** 10.1371/journal.pone.0091444

**Published:** 2014-03-10

**Authors:** Xuejian Wang, Hao Yu, Zhenhua Cai, Zhifeng Wang, Baojun Ma, Yi Zhang, Zi Ye

**Affiliations:** Department of Neurosurgery, the Second Hospital affiliated to Nantong University, Nantong University, Nantong, Jiangsu, China; Beijing Institiute of Otolaryngology, China

## Abstract

**Objective:**

To study anatomical structures related to Meckel cave with endonasal endoscopic approach and to provide an anatomical basis for endoscopic surgery in Meckel cave.

**Methods:**

Meckel cave of 5 adult skulls (10 sides) were fixed with 10% formalin. The anatomical structures of Meckel cave and the related zones adjacent to Meckel cave were observed and measured with endoscopic endonasal approach.

**Results:**

Endoscopic endonasal, endo-maxillary sinus, and endo-pterygoid process approaches were successfully applied in observation of the anatomical structures of meckel cave and the rerated zones adjacent to Meckel cave and in measurement of distances between related anatomical structures for each case of samples. The relevant data were obtained. The distance between the front mouth of palatovaginal canal and vidian canal was 21.4±7 mm, the distance between opening of sphenoid sinus to the upper margin of the choana was 22.3±2.8 mm, the distance between the opening of vidian and foramen rotundum was 7.57±0.7 mm and the length of the pterygoid canal was 13.3±1.2 mm. Based on these data, the positions of the related important structures can be roughly located during surgical operation and various important structures in Meckel cave and its adjacent zones can be found out in a convenient and safe way.

**Conclusion:**

1) It is feasible to use endonasal endoscopic approach to perform surgical operation in Meckel cave; 2) Use of endonasal endoscopic approach can protect and fully take the advantage of the vidian nerve to locate the position of foramina lacerum of the internal carotid artery during surgical operation; and 3) the observational and experimental data obtained with this approach can provide the rational basis for clinical operation procedures.

## Introduction

Since the anatomical concept ‘Meckel’ was firstly proposed by Meckel in 1832 [1], Meckel cave (also called Meckle cavity), a space beneath the dura matters-containing trigeminal ganglion, has been one of the most difficult anatomical regions to deal with for a long time due to its anatomical uniqueness, such as its deep position and meticulous structures, which are surrounded by other adjacent complex structures and as well as other reasons. Thus, it has been one of the technical difficulties to treat the space-occupying lesion specifically located in this region. Previously, the space-occupying lesion located in Meckel cave had been mainly treated with radiotherapy and craniotomy. During the last several decades, the endoscopic techniques have been gradually applied and extended, especially after the endonasal approach has been expanded, the advantages of endoscopic and surgical indications have received further recognition [2]–[7] and the application scope of endoscopic nasal approach has been gradually broadened. In the present study, we performed an anatomical analysis on Meckel cave of fresh human skulls perfused with red latex using endoscopic endonasal, endo-maxillary sinus, and endo-pterygoid process approach to investigate the anatomical characteristics of Meckel cave and the feasibility of using these approaches to study Meckel cave, aiming to provide anatomical basis for the application of endoscopic endonasal approach for the surgical treatment of the space-occupying lesions in Meckel cave.

## Materials and Methods

### 1. General Information about Preoperative Preparation

#### Ethics statement

The entire study was conducted according to the principles expressed in the Declaration of Helsinki. The Ethical Committee of Nantong University has specifically approved this study.

The specimens of fresh skulls of five human adults (10 sides) were provided by The Laboratory of Surgery and Anatomy of Nantong University. The exact ages and genders of these adults were unable to be determined in this study. These specimens (neck 2 or above) were preserved. No abnormal substances, newly formed substances, the obviously space-occupying lesions and structural changes in all specimens at sella area, sphenoid sinus, maxillary sinus, Meckel cave and cavernous sinus were seen. All specimens were perfused with red latex in the bilateral carotid artery and vertebral artery, and with blue latex in bilateral internal jugular vein, respectively. Ten dried specimens of human skulls were provided by The Laboratory of Surgery and Anatomy of Nantong University. Navigation was developed and made by Digital Center of Fudan University (Shanghai, China). The skull surgery fixation system, zero degree rigid endoscopy and light source, monitors, video recording system (Karl Storz, German), endoscopic surgical instruments, lead wire, and soft feet etc. were all made by us.

### 2. Experimental Methods

**Figure 1:**
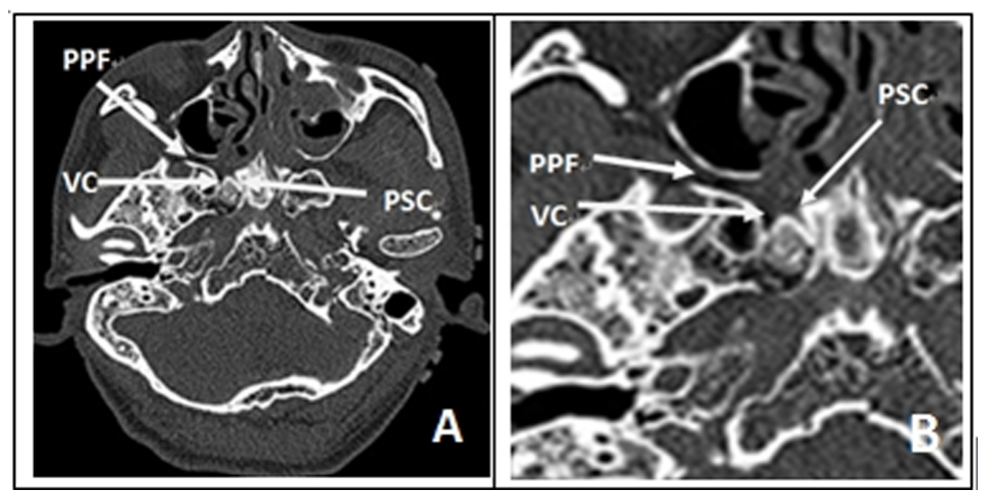
(A&B) Axial CT examinations. It showed relationship of foramen rotundum, vidian canal and palatosphenoidal canal (palatovaginal canal). (PPF: pterygomandibular fossa; PC: vidian canal; PSC: Palatosphenoidal Canal (Palatovaginal Canal); VC: vidian nerve).

**Figure 2:**
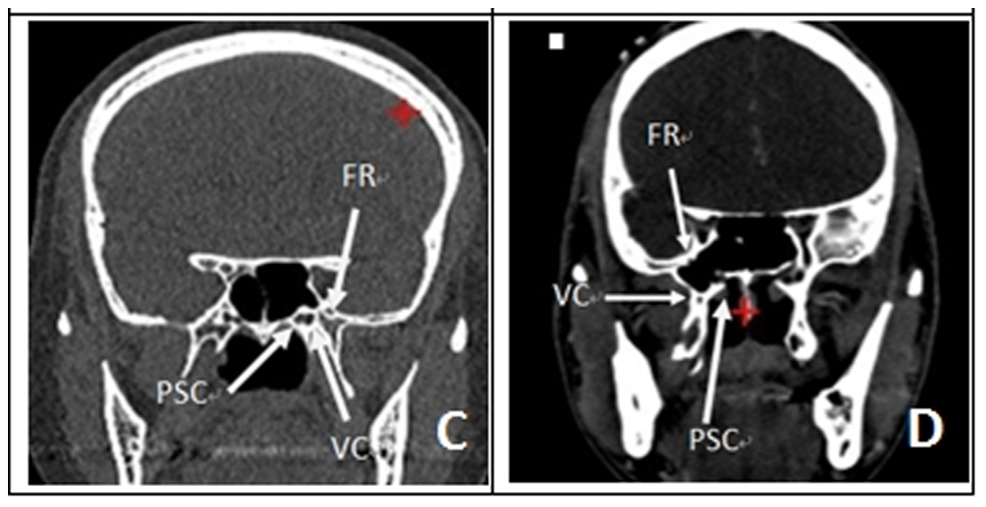
(C&D) Coronal CT examinations. it showed their relationship. (FR: foramen rotundum; PSC: Palatosphenoidal Canal (Palatovaginal Canal); VC: vidian nerve).

Each of the arteriovenous system of 5 intact human skulls (10 sides) derived from cadavers was perfused with red and blue latex, respectively. Before the experiment, CT navigation scan was carried out in all five skulls of cadavers (Figure 1 & Figure 2). Fresh specimens of skulls were thawed at room temperature (25°C) for 24 hours. The heads were set fade-away at an angle of 30 degree and were fixed with three screw brackets. Navigation was registered and verified, and errors were less than 2 mm. The nasal cavity was cleaned and the anatomic structures of nasal cavity were identified. The middle turbinate on the experimental sides was resected and the contralateral middle turbinate on the opposite side was relocated outwardly to expand the operational space (Figure 3.1). After transverse incision of mucosa at the attachment site of the lower and inferior edges of the middle turbinate on the experimental side, sieve ridge can be found. Processing along sieve ridge toward the superior and lateral margins, sphenopalatine (palatosphenoidal) artery and palatosphenoidal foramen could be found. Processing along the terminal of sieve ridge to its inner side, pharyngeal opening of palatovaginal canal with pharyngeal branch of maxillary artery out of skull base could be found. Once the bone wall of palatovaginal canal which covers the palatosphenoidal artery surface was removed, the contents of palatosphenoidal canal and posterior bone notches were exposed and could be seen (Figure 3.2). The anterior wall of sphenoid sinus and posterior ethmoid sinus were opened widely (Figure 3.3), the stoma at the opening mouth of frontal sinus was expanded toward the lateral margin, superior margin and lower margin, the nasal septum was returned back to the opening mouth of maxillary sinus, the lateral portion of the back wall of maxillary was removed and close to the infraorbital nerve, the contents of pterygopalatine fossa could be seen at this time. At the lateral portion of pterygoid canal, the contents of pterygopalatine fossa were pushed and moved outwardly, the position of the anterior aperture of vidian canal could be located (Figure 4.4). The opening of infraorbital (V2) nerve of foramen rotundum could be found when the lateral portion of pterygoid canal was moved outwardly. Bottom wall of sphenoidal sinus was abraded to the depth of central dival depression and downwardly to the lower margin of the frond mouth of vidian canal at the sphenoid floor. The outside boundary was where anterior aperture of vidian canal was located. A part of the bone at the bottom wall of sphenoidal sinus was abraded away. Through the navigation versification (Figure 1 & Figure 2), the bones of the lower margin of pterygoid canal were abraded. The location and depth of internal carotid artery could be identified and assessed, and the bones at the superior margin of pterygoid canal were removed. Finally, the thin layer of bone walls on the superior side, inner side and lower margin of vidian nerves were removed and vidian nerves were exposed (Figure 4.5). The vidian nerves were gently pushed and moved toward the superior margin. At this time, the bones outside the vidian nerves were exposed. The bones were abraded with an abrader and caution must to be taken while abrading the bones to prevent damages to pterygoid canal nerve. Abrading was processed directly from the lateral margin and to internal carotid artery and vidian nerve and finally to the lag of internal carotid artery. After removal of the front and lateral bones of internal carotid artery within this section, the region which was covered by the dura mater was exposed. At this time, internal carotid artery could be seen at its inner and lower sides whereas V2 and V3 branches could be seen at its lateral side. Once this dura mater was cut and opened, the anatomical structures such as gasserian ganglion and trigeminal nerve could be seen, i.e. it was the Meckel cave (Figure 4.6). At this time, further processing could lead to other regions such as petrous apex.

**Figure 3:**
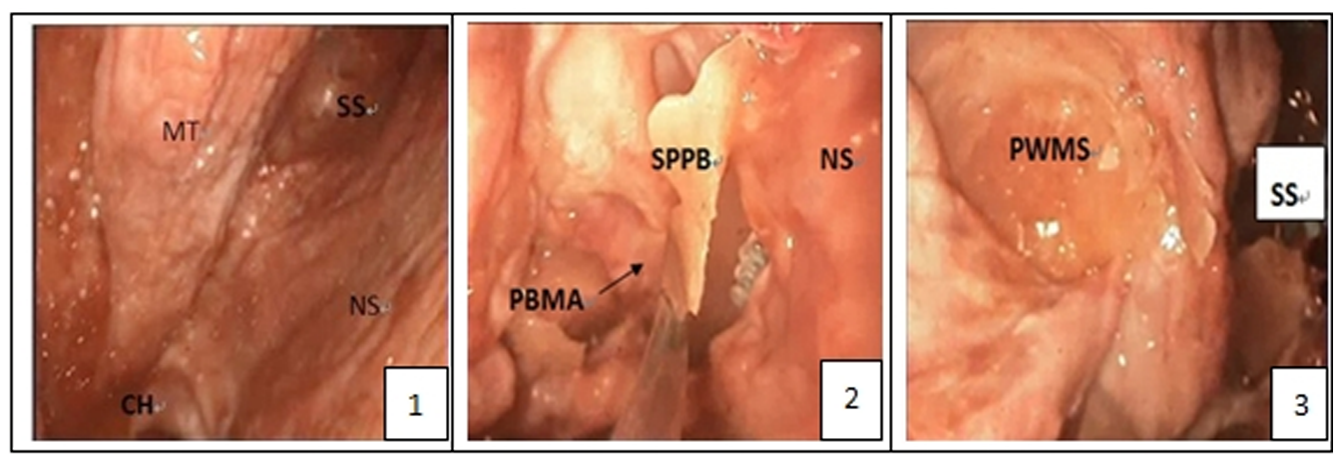
The anatomy step of Meckel Cave. Figure 3.1: The anatomy of nasal cave; Figure 3.2: after the removal of the front wall of palatosphenoidal canal (palatovaginal canal), pharyngeal branches of palatosphenoidal artery and pterygopalatine fossa contents were exposed; Figure 3.3: the posterior wall of the maxillary sinus. (CH:choana; MT: middle turbinate; NS: nasal septum; PBMA: the pharyngeal branch of the maxillary artery; PWMS: posterior wall of maxillary sinus; SPPB: the sphenoid process of the palatine bone; SS: sphenoid sinus;).

**Figure 4:**
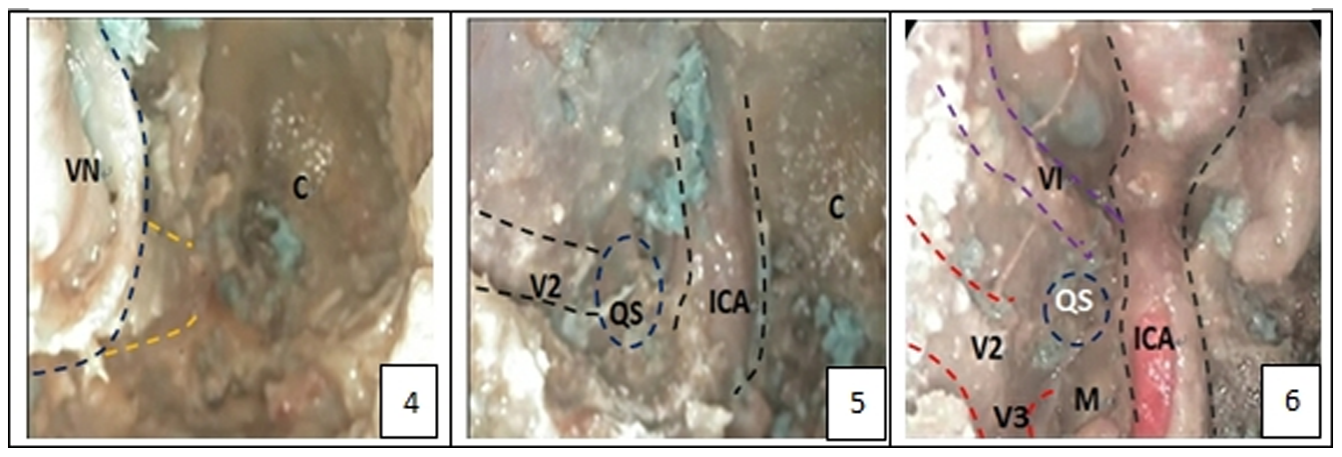
The anatomy step of Meckel Cave. Figure 4.4: the position of the front door of vidian canal and vidian nerve; Figure 4.5: after removal anterior and lateral part of the bone of Meckel cave, Meckel cave which was covered by the dura mater was exposed, the interior and inferior margin of which is the internal carotid artery, lateral side is V2 and V3 branches; Figure 4.6: Dura mater was resected, Meckel cave was opened. (C: clivis; ICA: internal carotid artery; M: meckel cave; QS: quadrilateral space; SS: sphenoid sinus; V: trigeminal nerve; V2: maxillary branch of the trigeminal nerve; V3: mandibular branch of the trigeminal nerve; VI: abducent nerve; VN: vidian nerve).

### 3. Data Processing

The observations and measurement of parameters related to the anatomical structures and distances of Meckel cave were observed and measured with endoscopic endonasal approach. All measurements were repeated three times and the averaged data were obtained. The measured value of the variable used was expressed as X ± SD (standard deviation).

## Results

Applications of endoscopic endonasal, endo-maxillary sinus, endo-pterygoid process approaches were found to be feasible for performing surgery in Meckel sac and these approaches were found to be capable of reaching and dealing with Meckel sac. During the experiment, the distance between the front mouth of palatovaginal canal and vidian canal was 21.4±7 mm, distance from opening of sphenoid sinusto the supperior margin of the choana was measured to 22.3±2.8 mm, the distance between the opening of vidian and foramen rotundum is was 7.57±0.7 mm and the length of the pterygoid canal was 13.3±1.2 mm. Based on the measured data, it is possible to roughly locate the position of the important anatomical structures during surgical operation and the important anatomical structures in both Meckel sac and its adjacent zones could be clearly found out in a convenient and safe way.

## Discussion

With the continuous development of endoscopic techniques, the indication and scope of endoscopic transnasal approach has expanded continuously. It has been developed from the initial classic endoscopic transsphenoidal pituitary tumor surgery to the anterior skull base, back to odontoid process, and lateral to skull base and has been widely applied in clinical practices. However, because the endoscopic techniques has been applied in in neurosurgery for a relatively short time, there are still a number of “blind spots” which require experimental and surgical researches, such as the studies on anatomical features of the Meckel sac, to further demonstrate and overcome. Currently, only a few studies on anatomical characteristics of this region with endoscopic techniques have been reported and scattered in the literature and even much fewer studies on the clinical applications of endoscopic techniques have been reported [2], [3], [8], [9].

Surgical operation in Meckel cavity has been one of the most difficult surgeries because this region is located in a deep place, its space is narrow and its surrounding anatomical structures and craniotomy are even more complicated. Before endoscopic techniques were put in use, craniotomy was more frequently used. Previously, clinicians had developed a number of craniotomy approaches specifically for different lesions, which can, in generally, be divided into anterolateral, lateral and posterolateralposter lateral approaches and etc. [10]–[14]. However, none of these surgical approaches can fully deal with surgical problems in this region because each of them has varying degrees of drawbacks, such as more trauma, more stretch in brain tissue, the impact to beauty and as well as many other shortcomings [15]. Additionally, for surgical treatment of the pathological lesions in Meckel region, the previously used surgical approaches have anatomical disadvantage without exception, it is particularly difficult to avoid tractions on the nerves and blood vessels, which causes high risks. Moreover, under the view with microscope into this region, surgical vision field was limited, which hindered the clinical application [2], [16]. For the above reasons, we carried out research on application of endoscopic transnasal, pterygoid approaches to visualize Meckel region based on the following major considerations and reasons: 1) during endoscopic nasal surgery, traction on the brain tissue is relatively small. For the previously used surgical methods, all of them are approached to Meckel sac region from the outside and thus, they inevitably need to stretch the brain tissue whereas the endoscopic nasal approach is to arrive Meckel sac region from anteromedial region and thus, the injuries caused by traction to trigeminal nerve and other neurological structures can be avoided; 2) Meckel sac is located in the inferior-external lateral of the cavernous sinus, the inner structure of which is trigeminal nerve, in which the commonly occurring pathological lesions are the trigeminal schwannomas and meningiomas [1], [17]–[22]. When the tumors are enlarged, Meckel cave can expand the its space which can be more convenient for performing surgical operation; 3) endoscopy light source system can provide direct lights to the surgical field, and can change light direction according to the requirements of the surgical field for resection of tumor tissue; 4) endoscopic technique has become more and more mature. The previous complications such as the problems of cerebrospinal fluid leakage now have received a better solution. Very mature endoscopic techniques have been applied in many parts of the world. Although it is a late start, this technology has been extended in our country at present as well. To our department, more than 400 cases of surgery has been performed through endoscopic nasal surgery and we have gained a considerable experiences in application and expansion endonasal surgical techniques for surgery on Meckel cavity. We already have quite mature skills and experiences in dealing with some cases of high flow cerebrospinal fluid leakage; 5) endonasal endoscopy is a minimally invasive technique with less postoperative discomfort, rapid recovery and yet have no negative effects on beauty and also has its unique advantages as compared to the previous craniotomy.

In this study, we also found that there are still some problems after performing endoscopic surgery in Meckel cave because this technique also has some disadvantages and can cause some problems. Clinical attentions and further research are needed s to resolve the following problems: 1) due to the exquisite anatomical features and complex nature of this region, it is extremely important that pre-operative design and pre-operative surgical procedures must be precisely planed and that the surgeons must be well familiar with the detailed anatomic structures of this region and the surgical operation must be performed by the experienced physician and tight fit with an assistant. Sabancı et al. had the similar point of view [1]. 2) Because this region is located closely to the internal carotid artery and some other important tissues/organs such as blood vessels, the trigeminal nerve and other cranial nerves, the endoscopic surgery can be performed only on the basis after conducting relevant endoscopic surgery and surgeons have gained proficiency and skills in endoscopic surgery, because even a slightest mistake can cause unpredictable risks; 3) endoscopy lens provide a tubular light source for surgical operation, any contaminations to endoscopy lens can affect visual field. Because anatomical structures of this region are exquisite, more attentions need to be paid when operation is performed in this region; 4) In general, intraoperative cerebrospinal fluid (CSF) leakage and exposure of internal carotid artery sometime occur when endoscopic surgery is performed in this region, further radiotherapy is needed for treatment for the recurrence of the diseases, such as the trigeminal nerve sheath tumors and other diseases. Thus, the previous general reconstruction has been inappropriate. Kassam proposed that nasal pedicled mucosal flap was an excellent pointer for reconstruction after such surgery. Currently, the reconstruction of the nasal pedicled mucosal flap technique has been successfully carried out in our department. The incidence of postoperative cerebrospinal fluid leakage was low, further enhancing the safety of conducting surgery in the region. In summary, while there are still some shortcomings of applying endoscopic nasal approach to Meckel surgery, all of the difficulties can be overcome after skilled and experienced endoscopy operation has been conducted.

In using this technology approach, we realized that the pterygoid canal nerve can be used to find out the internal carotid artery, to avoid the damages to it and, at the same time, to protect these nerves. The nerve of the pterygoid canal (Vidian nerve) is formed by the junction of the great petrosal nerve and the deep petrosal nerve within the pterygoid canal, which are the two types of nerves that contain fibres from sympathetic plexus branches of the internal carotid artery and parasympathetic branch of facial nerve. Damages to them can cause a decreased tear secretion and uncomfortable nasopharynx feeling. The injury to the first branch of trigeminal nerve can cause the cases of paresthesia in the ophthalmic corneal, which can more easily lead to corneal ulcers and even eventually to blindness. The consequences are serious and thus, protection is needed. There are three openings in the back wall of pterygopalatine fossa. They are foramen rotundum, the front door of vidian canal and palatovaginal canal. Among which, palatosphenoidal canal has been reported to be composited of external and inferior wall of the sphenoid sinus and sphenoidal process of palatine, and was renamed palatovaginal canal [23]. It has been reported the relative positions of these three openings, i.e. the front door of vadian canal and palatovaginal canal opening were oriented in a horizontal position whereas foramen rotundum was located at the lateral and superior sides of other two openings with vadian canal nerve being directed at the lap of internal carotid artery [24]. The lap of internal carotid artery exposed foramina lacerum and its position was converted from the horizontal position to the lateral position, whereby the front door of vadian canal can be found by palatovaginal canal and the lap of internal carotid artery can be looked for by the front door of vadian canal and vadian nerve. There have been related reports in the literature [9], [23], [25].

Due to the anatomical uniqueness of Meckel area, application of navigation and other new technologies are essential [26], [27]. Intraoperative real-time navigation verification and use of vascular Doppler ultrasound technique to monitor the blood vessels can effectively protect the pterygoid canal nerve, internal carotid artery and structures of other important tissues, and to avoid fatal danger. In this study, we used the navigation technology, which is useful to find out the front door of pterygoid canal and to locate the position of carotid artery, has played an importantly supporting role for the experiments and provided technical support for further surgery.

In term of the choice of surgical approach, we believed that the appropriate surgical approach should be chosen according to the specific circumstances of the targeted lesion. The advantages of endoscopic endonasal approach are: 1) it is minimally invasive and causes less brain tissue stretch and 2) it has obvious anatomical lesions advantage for lesions in the anterior medial of trigeminal nerve [2]. However, for those lesions that are mainly located in the middle cranial fossa epidural, inferior temporal epidural approach could be the appropriate choice [28]–[32]. For the main body that is located in the posterior fossa lesions, posterolateral approach is still the preferred choice. Therefore, preoperative examination, discussion and prices design of operational procedures are essential for the safety in performing the surgical operation in Meckel cavity.

In this study, we measured the distance between the front mouth of palatovaginal canal and vidian canal, distance from opening of sphenoid sinus to the upper margin of the choana, and the opening of vidian and foramen rotundum. These data are useful because they could have a supporting role for locating the position of the front mouth of vidian canal intraoperative. We also measured the length of the pterygoid canal, which could be useful for verification, looking for and protection of the internal carotid artery intraoperative.

## Conclusion

In summary, when the endoscopic endonasal approach is applied to perform surgical operation in Meckel area, pterygoid canal nerve is one of the most important anatomical structures and thus plays a very important role for surgical operation in this region. Therefore, being completely familiar with the precise position of pterygoid canal nerve and its relationships with the surrounding anatomical structures such as internal carotid artery is extremely important for helping and guiding to perform surgical operation with endoscopic endonasal approach for the tumor present in Meckel sac region. Endoscopic transnasal approach to Meckel region can well reveal the space-occupying lesions in Meckel area, particularly within the area under the front Meckel region. This surgical approach has obvious advantages over the previously used surgical approaches. However, due to the relatively later application and extension of this approach in our country, the neurosurgeons/physicians are still not quite familiar with this surgical approach. Furthermore, the surgical operation in this region is relatively risky and complex, in order to perform the surgical operation well in this region, neurosurgeons/physicians must have mastered endoscopic techniques and must be well familiar with endoscopic anatomy of this region and the surrounding tissues and, at the same time, use navigation and other new technologies for help and guide.
